# Excitotoxic lesions of the infralimbic, but not prelimbic cortex facilitate reversal of appetitive discriminative context conditioning: the role of the infralimbic cortex in context generalization

**DOI:** 10.3389/fnbeh.2014.00063

**Published:** 2014-02-28

**Authors:** Rachel Ashwell, Rutsuko Ito

**Affiliations:** ^1^Department of Experimental Psychology, University of OxfordOxford, UK; ^2^Department of Psychology, University of Toronto ScarboroughToronto, ON, Canada

**Keywords:** spatial context learning, reversal learning, medial prefrontal cortex, prelimbic cortex, infralimbic cortex, conditioned place preference, context generalization

## Abstract

The prelimbic and infralimbic regions of the rat medial prefrontal cortex (mPFC) are important components of the limbic cortico-striatal circuit, receiving converging projections from the hippocampus (HPC) and amygdala. Mounting evidence points to these regions having opposing roles in the regulation of the expression of contextual fear and context-induced cocaine-seeking. To investigate this functional differentiation in motivated behavior further, this study employed a novel radial maze task previously shown to be dependent on the integrity of the hippocampus and its functional connection to the nucleus accumbens (NAc) shell, to investigate the effects of selective excitotoxic lesions of the prelimbic (PL) and infralimbic (IL) upon the spatial contextual control over reward learning. To this end, rats were trained to develop discriminative responding towards a reward-associated discrete cue presented in three out of six spatial locations (3 arms out of 6 radial maze arms), and to avoid the same discrete cue presented in the other three spatial locations. Once acquired, the reward contingencies of the spatial locations were reversed, such that responding to the cue presented in a previously rewarded location was no longer rewarded. Furthermore, the acquisition of spatial learning was probed separately using conditioned place preference (CPP) and the monitoring of arm selection at the beginning of each training session. Lesions of the PL transiently attenuated the acquisition of the initial cue approach training and spatial learning, while leaving reversal learning intact. In contrast, IL lesions led to a significantly superior performance of spatial context-dependent discriminative cue approach and reversal learning, in the absence of a significant preference for the new reward-associated spatial locations. These results indicate that the PL and IL have functionally dissociative, and potentially opposite roles in the regulation of spatial contextual control over appetitive learning.

## Introduction

The mammalian prefrontal cortex has been implicated in a diverse range of cognitive functions that reflects its importance in the optimization and allocation of resources to meet changing task demands and rules (Euston et al., 2012). Its pattern of afferent and efferent connectivity indicates that the prefrontal cortex is uniquely positioned to integrate information from the sensory, limbic and autonomic systems, to exert executive control over motor, cognitive and autonomic functions via topographical connections with the striatum (Alexander and Crutcher, 1990; Groenewegen and Uylings, 2000; Chudasama and Robbins, 2006; Hoover and Vertes, 2007). The ventral prelimbic (PL) and infralimbic (IL) regions of the rat medial prefrontal cortex (mPFC) are components of the “affective” cortico-striatal loop, which receive converging, excitatory inputs from the hippocampus (HPC) and basolateral nucleus of the amygdala (BLA; Ishikawa and Nakamura, 2003).

Consistent with their close anatomical links with the HPC, a large body of lesion evidence supports the notion that these areas of the mPFC support HPC-mediated learning. Large mPFC lesions that encompass the anterior cingulate region, as well as the PL and IL have been shown to impair radial maze and water maze performance, which require the flexible use of multiple distal (spatial) cues to guide navigation (Kolb et al., 1983; Compton et al., 1997). More selective lesions targeting the PL-IL region have implicated these particular areas in spatial and object working memory but not in spatial discrimination (Ragozzino et al., 1998). Selective temporary inactivation of the PL region also impairs performance on a delayed spatial win-shift task, due to deficient retrieval of trial-unique information (Seamans et al., 1995). Floresco et al. (1997) obtained the same pattern of results with an asymmetric disconnection inactivation between ventral Cornu Ammonis 1 (CA1)/subiculum and the PL, supporting a critical role for the PL in accessing spatial information stored in the HPC. However, others have failed to observe deficits in spatial learning, arguing instead that previous reports of deficits in spatial tasks following mPFC lesions may have reflected impaired egocentric or response learning, rather than spatial learning (De Bruin et al., 2001; Lacroix et al., 2002; Deacon et al., 2003). Furthermore, spatial working memory deficits associated with damage to the PL-IL region are difficult to separate from concomitant attentional, or behavioral flexibility deficits (Delatour and Gisquet-Verrier, 2000; Boulougouris et al., 2007).

The mPFC has also been implicated in contextual control over motivated behavior, namely in context-induced reinstatement of drug-seeking (Peters et al., 2009; Bossert et al., 2011), expression of contextual fear (Corcoran and Quirk, 2007) and the encoding of contextual representations (Hyman et al., 2012). Furthermore, increasing evidence highlights the importance of considering different subregions of the mPFC in subserving functionally distinct and even opposing roles in contextual processing, along the dorsal (anterior cingulate + PL cortex)-ventral (IL cortex) axis. Thus, the dorsal mPFC has been linked to the inhibition of the reinstatement of context-induced cocaine-seeking and expression of conditioned fear, while the ventral aspect has been associated with driving cocaine-seeking and expression of conditioned fear (Vidal-Gonzalez et al., 2006; Peters et al., 2008), albeit not all data are consistent with this proposed dichotomy (Bossert et al., 2011).

The present study sought to further investigate the functional dichotomy of the mPFC in contextual control over appetitively motivated behavior, by employing a novel task to investigate the effects of selective excitotoxic lesions of the PL and IL in: (1) the acquisition of spatial contextual control over appetitive approach behavior, previously found to be dependent on the integrity of the HPC and nucleus accumbens (NAc) shell (Ito et al., 2008); (2) spatial discrimination (measured by conditioned place preference (CPP) test), also known to depend upon the HPC and NAc shell (Ito et al., 2008); and (3) spatial context reversal learning, as a measure of behavioral flexibility.

## Materials and methods

### Subjects

Subjects were 25 male Lister Hooded Rats (Charles River Ltd, UK) weighing between 330 and 400 g at the time of surgery. They were group-housed in a room held at a temperature of 21°C under a 12 h light/dark cycle (lights off 7 P.M.). Following recovery from surgery, water was available *ad libitum* and food (laboratory chow, Purina) was restricted to 20 g laboratory chow/day, sufficient to maintain 90% pre-operative body weight. All experiments were carried out during the light phase, between 08:00 and 18:00 h and in accordance with the United Kingdom 1986 Animals (Scientific Procedures) Act Project License no. 30/2561.

### Surgical procedures

Rats were divided into three groups (8 shams, 8 PL lesions, 9 IL lesions). In all surgical procedures, animals were anesthetized with isoflurane (Abbott Farm, UK), and placed in a stereotaxic frame with the incisor bar set at −3.3 mm below the interaural line. A 1 µl SGE syringe (SGE, Baton Rouge, USA) was then lowered into either the PL or IL, and the neurotoxin quinolinic acid (0.09 M, Sigma-Aldrich, UK) was infused bilaterally at the following co-ordinates: PL: 0.3 µl each at (1) AP = +3.8, L = ±0.8, D/V (dura) = −3.0, (2) AP = +2.8, L = ± 0.8, D/V = −3.3, IL: 0.2 µl each at (1) AP = +3.0, L = ± 0.7, D/V = −4.5, (2) AP = +2.5, L = ±0.7, D/V = −4.5. The sham control group was treated identically to the lesion groups, except that they received injections of sterile phosphate buffer (sterile PB), instead of the toxin. Following surgery, rats were allowed a recovery period of at least 7 days prior to behavioral testing, with food available *ad libitum*.

### Behavioral apparatus

All behavioral testing took place in a purpose-built automated 6-arm radial maze apparatus (Medical Associates), placed on a rotatable table elevated 1 m above the floor. The maze consisted of a central, white Polyvinyl chloride (PVC) floored compartment in the shape of a regular hexagon, and six connecting arms identical in size (46 cm (L) × 16.5 cm (H) × 9 cm (W)) and other physical features. An electronically-operated guillotine door served as the entrance to each arm. Arms were enclosed by transparent Plexiglass to allow rats visual access to extra-maze cues. At the end of each arm was a receding well consisting of a tray into which sucrose could be delivered, a 1.8 W, 17 V well light, and nose-poke sensor. Three of the wells were connected to their own individual infusion pumps placed outside the apparatus. The arms had stainless steel grid floors consisting of 5 mm rods and two sets of infrared beams were located 2 cm, and 3 cm away from the entrance to monitor animals’ entry and exit into the arms. Each arm was covered with a transparent Plexiglas lid, to allow recording of behavior via a video camera mounted above the apparatus (Microsoft, USA). The apparatus was controlled and behavioral data collection achieved using MED-PC version IV.

The apparatus was situated in a testing room containing a number of extra-maze objects, which remained in the same position in the room for the duration of the experiment. The floor and walls of the apparatus were wiped down with ethanol solution following each session to eliminate any odor traces. At the end of each training day the apparatus was rotated by 60, 120 or 180° clockwise or anti-clockwise, in a randomized order, to ensure that conditioning to intra-maze cues was minimized.

### Behavioral procedures (Figure 1)

#### Habituation

All rats were given one 15-min habituation session, in which they were free to explore all 6 arms. 0.2 ml of 20% sucrose solution was placed into all wells to encourage rats to learn to expect a reward at the end of the arms.

**Figure 1:**
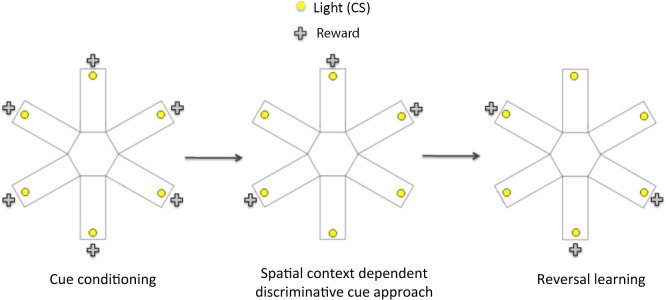
**Schematic diagram showing the three phases of the spatial context dependent cue learning and reversal learning task.** In the cue conditioning phase, animals were trained to learn that responding to a flashing light cue (conditioned stimulus, CS) within 15 s of its onset led to a sucrose reward. Once the rats reached criterion performance on cue conditioning, they then underwent spatial context- dependent discriminative cue learning, in which rats were trained to develop discriminative approach response to a CS presented in three rewarded locations (arms), and to withhold their response to a CS presented in three non-rewarded locations. Following the acquisition of discriminative cue approach, rats were tested on a conditioned place preference (CPP) test to establish if they had learnt the reward-associated locations. In the final phase (reversal learning), rats were trained to respond to CS presented in previously non-rewarded locations to obtain reward. At the end of reversal learning, they were given another CPP test to assess the acquisition of the new reward-associated locations.

### Cue conditioning (sessions 1–4)

During this phase, rats were trained to approach a 15 s flashing light stimulus presented at the end of an arm, to obtain 0.25 ml sucrose solution delivered to a well positioned directly under the light stimulus (CS). Each daily session consisted of rats receiving 30 CS presentations. Upon the rat nose-poking in the well within 15 s of the CS onset, the sucrose reward (US) was delivered, and the response was recorded as a correct response. To facilitate learning, the 30 CS presentations took place in only three out of the six arms (with the doors to the non-rewarded arms closed), with a different combination of rewarded arm locations each day, the order of which was counterbalanced across rats (e.g., Session 1: arms 1, 3, 5; Session 2: arms 2, 4, 6; Session 3: arms 1, 2, 3; Session 4: arms 4, 5, 6). For each session, the total number of CS presentations in each arm was equal (*n* = 10) but the order of CS presentations was randomized across arms. Each trial could only be initiated by the first photobeam interruption in an arm that was not rewarded in the previous trial, so as to encourage exploration of all arms during the training session. The session terminated at the end of the 30th trial, and typically lasted 30 min. A criterion of 24 correct responses (80%) was used to indicate acquisition of the CS-US association. All rats were given four sessions and those that had not reached criterion performance by the fourth session were given additional sessions. This ensured that all rats acquired the CS-US association, which was a prerequisite for the next phase of training.

### Spatial context-dependent discriminative cue learning (sessions 5–12)

In this part of the experiment, rats had access to all six arms and were trained to learn that approaching CSs presented in three of the six arm (spatial) locations would lead to sucrose reward presentation, while responding to CSs presented in the other three arm locations had no consequence. Three rewarded and three non-rewarded arm locations were assigned for each rat, and remained the same across all subsequent sessions. A total of 8 sessions were given, with each session consisting of 30 CSs presented in random order, but in equal numbers, in each of the six locations. If the rat approached the light within the 15 s interval and the arm location had been pre-assigned as the “rewarded location”, sucrose was delivered. The number of approaches to the CS presented in the rewarded locations (CS+) and to the CS presented in the non-rewarded locations (CS−), as well as the latency of responses, were recorded for each session.

### Conditioned place preference test 1 (session 13)

Rats were given 5 min to explore the entire apparatus in the absence of the CS and sucrose presentations. The time spent and the number of nose-pokes made in each arm was recorded.

### Reversal learning (sessions 14–19)

Rats were trained to approach CSs presented in the three previously “non-rewarded” arm locations and to avoid approaching the arms in previously “rewarded” locations. The procedure was identical to that of discriminative spatial context training, except that sucrose was delivered only for responding to CSs presented in previously non-rewarded arm locations and the rats received a total of six sessions.

### Conditioned place preference test 2 (session 20)

Once more, rats were given 5 min to explore the entire apparatus in the absence of the CS and sucrose presentations. The time spent and the number of nose-pokes made in each arm were recorded.

### Data analysis

All data were analyzed using the SPSS statistical package version 19.0 (SPSS, Chicago, IL). The total number of correct trials recorded in each session during cue conditioning was subjected to repeated measures analysis of variance (ANOVA) with lesion group as the between-subjects factor (lesion: sham, PL and IL) and session as the within-subjects factor. Data obtained during the spatial context-dependent discriminative cue learning phase consisted of the number of approaches to CS+ and CS− for each session. We also calculated a difference score by subtracting the number of approaches to the CS− from that to the CS+ for each session. These data were subjected to repeated measures ANOVAs with lesion group as the between-subjects factor (lesion: sham, PL and IL) and CS (CS+/CS−) and session as the within-subject factors. The same repeated measures ANOVAs were conducted for data recorded during reversal learning. Significant interactions were followed by tests of simple effects and multiple pairwise comparisons. For the CPP tests, two-tailed paired *t*-tests were conducted to compare the time spent exploring the reward-paired arms, and the non-rewarded arms in each lesion group.

## Results

### Histological analysis

**Figure 2:**
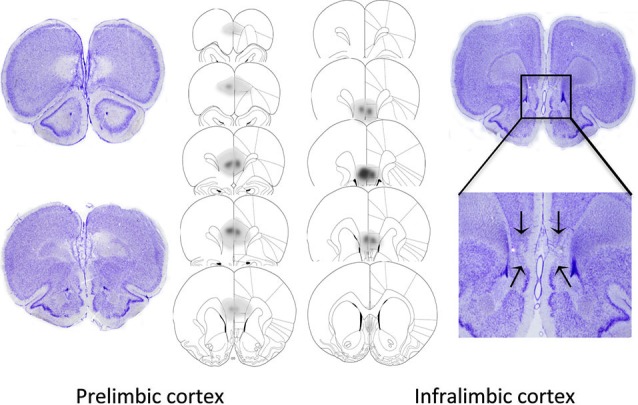
**Representative photomicrographs and extent of quinolinic lesions of the PL cortex (left) and IL cortex (right)**. Photomicrographs show cresyl violet-stained coronal sections of rat brains with selective PL, or IL lesions. PL lesions were typically marked by a complete loss of cell bodies in the PL area and preservation of neurons in the IL region. IL lesions typically induced marked shrinkage of surrounding tissue, while preserving the PL region. The schematic diagrams denote the largest (dark gray), and smallest (light gray) extent of neuronal damage in the PL and IL lesioned animals.

The extents of the excitotoxic lesions of the PL and IL are shown in Figure 2, based on the cytoarchitectonic borders taken from Paxinos and Watson (1998). Highly stringent criteria were followed for inclusion in data analysis to ensure selectivity of the damage to the target area (PL or IL). Thus, two rats were excluded from the PL group as their lesion extended to the IL region. Data from another rat was removed for sustaining a large unilateral lesion to the PL. The remaining 5 rats showed discrete bilateral lesions of the PL area that extended from bregma +4.40 mm to bregma +2.20 mm. One animal from the IL group was excluded from data analysis on the basis of a large lesion that extended caudally into the lateral septum. The remaining 8 animals showed discrete bilateral damage to the IL region extending from +3.2 mm to +2.2 mm from bregma, with complete neuronal loss and subsequent shrinkage of surrounding tissue. The lesion was occasionally found to encroach dorsally into the dorsal peduncular cortex, but in most cases, this was a unilateral encroachment.

### Cue conditioning

**Figure 3:**
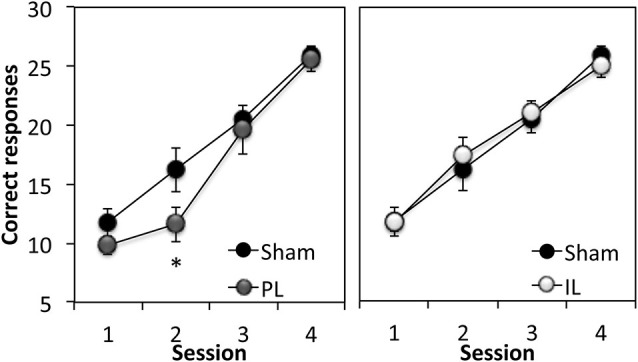
**Acquisition of discrete cue conditioning**. Mean ± SEM performance of sham-operated controls and prelimbic (PL—left panel) or infralimbic (IL—right panel) lesion groups in discrete cue conditioning, expressed as the number of “correct” responses made out of 30 trials in each session. A “correct” response was defined as responding to the CS within 15 s of its onset. * *p* < 0.05 compared to sham performance.

The first stage of the experiment assessed rats’ ability to respond to a 15 s flashing light stimulus (CS) in order to obtain a sucrose reward. A two-way ANOVA comparing the total number of correct responses (out of 30 trials) across four sessions in the sham group and PL group (Figure 3) revealed significant learning taking place in both groups (Session *F*_(3,30)_ = 50.10, *p* < 0.0001). However, the overall level of correct responses was significantly reduced in the PL group, compared to that in the sham group (Lesion effect: *F*_(1,10)_ = 4.75, *p* < 0.05), with a significantly attenuated performance of PL-lesioned rats on day 2 (*p* < 0.05). A two-way ANOVA comparing the acquisition of cue learning in the sham and IL groups (Figure 3) revealed that there was no difference in the overall level of correct responses, or pattern of acquisition (Session, *F*_(3,42)_ = 48.58, *p* < 0.0001) across the four sessions of cue conditioning between the sham and IL groups (no Lesion effect, *F*_(1,14)_ = 0.01, *p* = 0.91; no Lesion × Session interaction, *F*_(3,42)_ = 1.35, *p* = 0.12).

In summary, the acquisition of cue conditioning was selectively attenuated in the PL-lesioned rats compared to sham-operated rats, however, this effect was transient, as the PL-lesioned animals reached criterion performance at the same rate as the sham and IL groups (Trials to criterion: Sham; 127.5 ± 12, PL; 126 ± 6, IL; 131.25 ± 7.9, *F*_(2,20)_ = 0.12, *p* = 0.89).

### Spatial context-dependent discriminative cue learning

**Figure 4:**
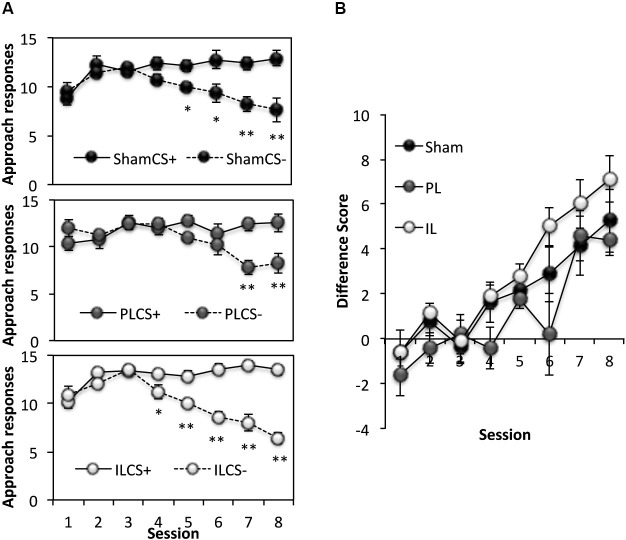
**Acquisition of spatial context-dependent discriminative cue learning. (A)** The mean ± SEM performance in the acquisition of spatial context-cue learning after sham, prelimbic (PL) and infralimbic lesions (IL), expressed as number of approaches to CS+ (rewarded) and CS− (non-rewarded) in each session. * *p* < 0.05, ** *p* < 0.01 compared to approach responses to CS+. **(B)** The acquisition data expressed as a difference score (number of approaches to CS+ (−) number of approaches to CS−).

This phase of the experiment assessed the ability of rats to use spatial information to acquire discriminative approach behavior towards a CS+, and away from CS− (Figure 4). Thus, rats learned to respond to a CS only when it was presented in specified rewarded locations (3 out of 6 arms). A three-way ANOVA of the number of responses to the CS+ and CS− across eight acquisition sessions (Figure 4A) revealed significant main effects of Session (*F*_(7,126)_ = 10.10, *p* < 0.0001), and CS (*F*_(1,18)_ = 56.19, *p* < 0.0001), as well as a significant Session × CS interaction (*F*_(7,126)_ = 56.14, *p* < 0.0001) indicating successful acquisition of spatial context-dependent discriminative cue learning in all groups, with a progressive increase in the number of approach responses to the CS+ (CS+; Session effect *F*_(7,126)_ = 8.33, *p* < 0.001), coupled with a decline in the number of approach responses to the CS− (Session effect; *F*_(7,126)_ = 18.95, *p* < 0.001). There was, however, a significant CS × Lesion group interaction (*F*_(2,18)_ = 3.58, *p* < 0.05). Further analyses revealed this effect to be attributable to a significant difference in the pattern of acquisition of spatial context-cue learning between the PL and IL groups (CS × Lesion interaction; *F*_(1,11)_ = 10.93, *p* < 0.01), with the IL group showing enhanced discriminative approach behavior overall (simple main effect of CS, *F*_(1,11)_ = 69.7, *p* < 0.0001), compared to the PL group (simple main effect of CS, *F*_(1,11)_ = 5.68, *p* < 0.05). This difference in the acquisition pattern of spatial context-cue learning in the IL and PL groups was confirmed by an ANOVA conducted on the difference score ([No of approaches to CS+] − [No of approaches to CS−], see Figure 4B), with significantly reduced differences scores across the eight sessions in the PL group, compared to the IL group (Lesion effect: *F*_(1,11)_ = 10.93, *p* < 0.01).

In summary, although there were no significant differences between the performance of the PL- or IL-lesioned rats and sham-operated rats in the number of correct responses to CS+ and CS− made across eight sessions, the IL-lesioned rats showed enhanced acquisition of spatial context-dependent discriminative cue approach behavior compared to the PL-lesioned rats.

### Spatial learning

#### Acquisition of spatial discrimination

**Figure 5:**
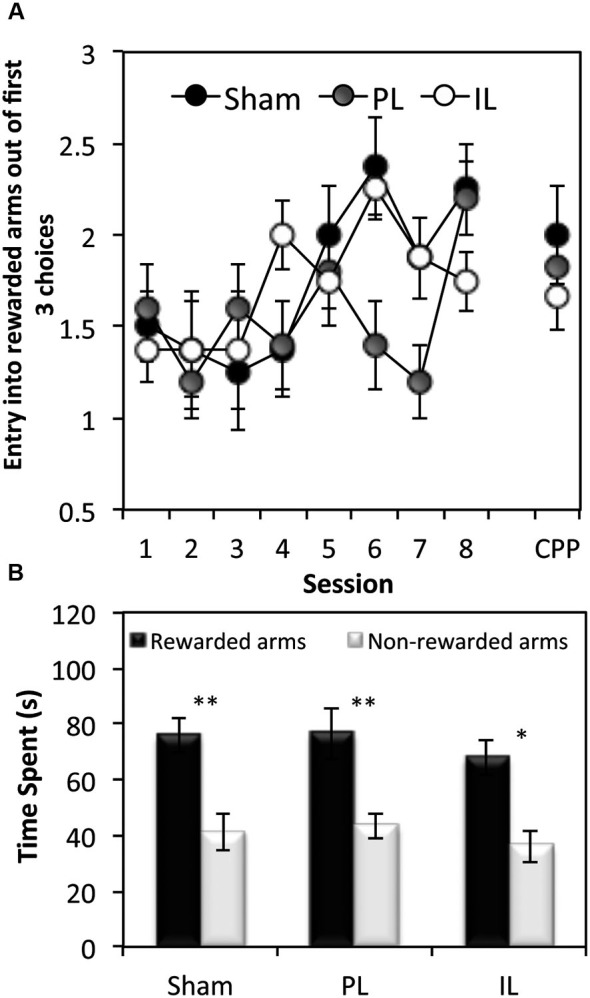
**Tests of spatial learning.** **(A)** Spatial reference memory test—the mean ± SEM number of arm entries out of the first 3 entries that were made into rewarded arms at the beginning of each session (and the conditioned place preference (CPP) test session), before the presentation of the CS. **(B)** Conditioned place preference test, expressed as mean ± SEM total time spent in the 3 rewarded or 3 non-rewarded arms (within a 5 min session) for sham controls, IL and PL - lesioned groups. * *p* < 0.05, ** *p* < 0.01 difference between time spent in rewarded vs. non-rewarded arms.

In order to assess the acquisition of spatial discrimination over the course of 8 training days, the first three choice of arm entry prior to the presentation of CSs was recorded at the start of each session of the context-dependent discriminative cue learning (Figure 5A). A two-way ANOVA of the number of entries into rewarded arms out of the first three choice of entry for all lesion groups revealed a significant main effect of Session (*F*_(7,126)_ = 4.27, *p* < 0.0001) indicating that all groups showed a progressive increase in rewarded arm entry across sessions. However, there was a significant Lesion effect (*F*_(2,18)_ = 3.45, *p* < 0.05) which was due to the overall choice for the rewarded arms being reduced in the PL group compared to the sham group (*p* < 0.05).

### Conditioned place preference test 1

Paired *t*-tests revealed all three groups to show a significant preference for rewarded arm locations in the absence of CS and reward presentations, as measured by the time spent (Figure 5B) in rewarded arms over non-rewarded arms (Sham: *t*_(7)_ = 3.55, *p* < 0.01, PL: *t*_(4)_ = 5.53, *p* < 0.01. IL: *t*_(7)_ = 3.40, *p* < 0.02).

In summary, while all groups acquired spatial-reward learning by the end of eight discriminative spatial context-cue learning sessions, the data on the first three choice entries at the beginning of the training session revealed the PL group to have been significantly impaired in entering reward-associated spatial locations.

### Reversal learning

**Figure 6:**
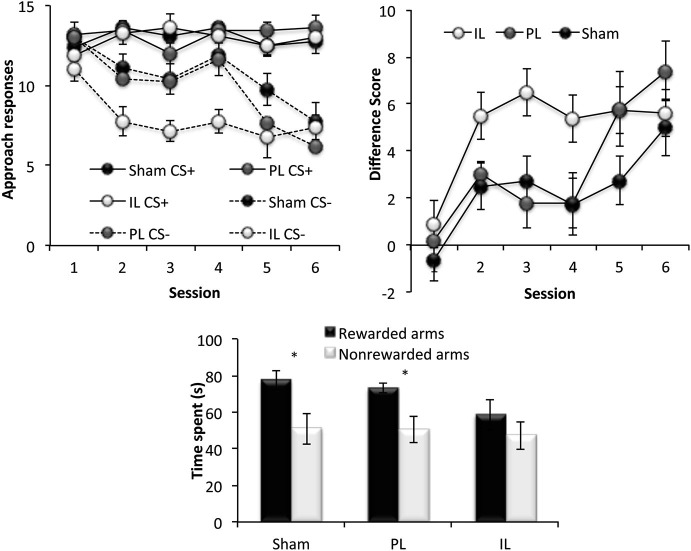
**Reversal learning.** The top two panels show the reversal learning performance of the sham, prelimbic (PL) and infralimbic lesion (IL) groups, expressed as the mean ± SEM number of approaches to CS+/CS− for each session (left) and the mean ± SEM difference score (right) for each session. The lower panel shows the second conditioned place preference test expressed as the mean ± SEM time spent in the 3 rewarded or 3 non-rewarded arms in a 5 min session. * *p* < 0.05, ** *p* < 0.01 difference between time spent in rewarded vs. non-rewarded arms.

This phase of the experiment assessed reversal learning; the ability to inhibit approach to CS presented in previously rewarded locations and to instead, respond to CS presented in previously non-rewarded locations (Figure 6). A three-way ANOVA for the number of responses to CS+ and CS− under new reward contingencies across six sessions revealed significant main effects of Session [*F*_(5,90)_ = 11.49, *p* < 0.001] and CS [*F*_(1,18)_ = 94.65, *p* < 0.001], as well as a significant Session × CS interaction (Session × CS [*F*_(5, 90)_ = 8.42, *p* < 0.001]), indicating successful reversal learning in all lesions groups, with responses to CS− decreasing considerably across sessions. There was, however a significant Lesion × CS interaction [*F*_(2,18)_ = 4.46, *p* < 0.03], and a main effect of Lesion approaching significance [*F*_(2,18)_ = 2.68, *p* = 0.09]. Further ANOVAs comparing the performance of lesion groups revealed that this effect was attributable to a significant Lesion × CS interaction [*F*_(1,14)_ = 7.86, *p* < 0.02] and an almost significant Lesion effect (*F*_(1,11)_ = 4.37, *p* = 0.055) between sham-operated and IL-lesioned rats, as well as a near significant CS × Lesion group interaction between the IL and PL groups [*F*_(1,11)_ = 4.55, *p* = 0.056]. Subsequent simple effects analyses revealed a significant main effect of Lesion (sham vs. IL-lesioned group) on the number of responses to the CS− [*F*_(1,14)_ = 7.57, *p* < 0.02], but not to CS+ [*F*_(1,14)_ = 0.04, *p* = 0.86].

### Conditioned place preference test 2

Paired *t*-tests revealed that while the sham and PL groups showed significant preference for the rewarded arms over non-rewarded arms (Sham: *t*_(7)_ = 3.42, *p* < 0.02, PL: *t*_(4)_ = 2.75, *p* < 0.05), the IL group did not show significant preference for the rewarded arms (IL: *t*_(7)_ = 1.44, *p* = 0.19).

In summary, all rats acquired reversal learning, but the IL-lesioned rats were significantly facilitated in doing so, due to IL-lesioned rats making significantly fewer responses to CS presented in non-rewarded locations, compared to sham-operated, and PL-lesioned rats. However, despite the facilitated acquisition of reversal learning, IL-lesioned rats failed to demonstrate CPP for new reward-associated spatial locations.

## Discussion

The present study presents novel findings of dissociative roles of the PL and IL mPFC in spatial context-dependent discriminative cue learning, and the reversal of established spatial context-dependent cue-reward contingencies. Selective, excitotoxic lesions of the PL transiently attenuated the acquisition of instrumental cue learning, and also attenuated the acquisition of spatial learning while leaving reversal learning intact. In contrast, selective, excitotoxic lesions of the IL had no effect on the acquisition of cue learning, context-dependent discriminative cue learning, or reversal learning. Instead, IL lesions led to a significant facilitation of reversal learning, despite the notable absence of CPP for the new reward-associated spatial location. These results indicate that the PL and IL have functionally dissociable roles in the regulation of contextual control over appetitive learning, and may even have opposing roles in some aspects of this behavioral control.

### Effect of infralimbic (IL) lesions on spatial context-dependent reversal learning

The major finding of the present study was the marked facilitation of spatial context-dependent reversal learning in IL-lesioned rats. This effect was primarily driven by IL-lesioned rats making fewer responses to the CS− (previously CS+), which could also be interpreted as the IL-lesioned rats making significantly fewer perseverative responses than sham-operated rats. One account for this finding is that the IL-lesioned rats were able to adapt their responding to the new reward contingencies more quickly because their performance was predominantly under the control of goal directed action-outcome associations, than that of the sham control rats whose behavior may have been under the control of stimulus-response associations by the time the reversal was implemented. Indeed, it has previously been shown that selective excitotoxic IL lesions prevent the transition of instrumental responding to habitual responding, such that performance remains goal-dependent even after substantial over-training (Killcross and Coutureau, 2003). This effect was in contrast to the effects of selective PL lesions, which led to a profound disruption in the acquisition of goal-directed responding (Killcross and Coutureau, 2003).

A further account of the enhanced reversal of spatial context-dependent cue reversal learning in IL-lesioned rats may be that IL lesions enhanced spatial/contextual control over conditioned responding. Of great pertinence to this idea is the finding of Rhodes and Killcross (2007) who found that selective excitotoxic lesions of the IL enhanced renewal of conditioned responses (CR-magazine approach) in the acquisition context (A), when the CR was extinguished in another context (B), but not when the rats were tested in the extinction context (B). Thus, IL lesions increased the context dependency of extinction, indicating that an intact IL serves to increase context generalization, by attenuating the context specificity of extinction. In the present study, it is conceivable that IL-lesioned rats were able to show enhanced discriminative conditioned responding to the CS both during the initial acquisition of spatial context-dependent discriminative cue learning and in its reversal due to a reduction in generalization of spatial locations or “contexts” defining the reward contingencies. The increased context-dependency in IL-lesioned rats potentially has superior explanatory power to the habit formation deficit account, as it can also explain the absence of CPP for the new reward-associated spatial location following reversal training. Thus, IL-lesioned rats would have learnt in the first CPP test that the test session represents an “extinction context” that is distinct from the spatial context-dependent cue learning context, marked by an absence of CS and US presentations. On subsequent (second) exposure to the “extinction context”, IL-lesioned rats showed enhanced contextual control (suppression) over conditioned responding, leading to a loss of expression of CPP. Indeed, the IL-lesioned rats appeared to spend more time in the central hub during the second CPP test, as if they were waiting for the appearance of the light cue CS. This potential role of the IL in the suppression of context-specificity is ecologically and behaviorally relevant, particularly in situations when diminishing the influence of contextual information upon decision making is more beneficial for an animal (Freidin and Kacelnik, 2011).

### Effect of prelimbic (PL) lesions on discrete cue conditioning and spatial context dependent cue learning

The transient attenuation of cue learning in PL-lesioned rats is consistent with previous reports of an impairment in the acquisition of appetitive instrumental learning, following discrete excitotoxic lesions of the PL (Corbit and Balleine, 2003) and NMDA receptor and/or dopamine D1 receptor blockade in the PL (Baldwin et al., 2002). Previous studies have shown PL-lesioned rats to be insensitive to outcome-specific devaluation (Corbit and Balleine, 2003; Killcross and Coutureau, 2003), in the absence of any debilitating effect on the acquisition of pavlovian approach responses, or the ability of pavlovian stimuli to have an excitatory impact on instrumental performance (Corbit and Balleine, 2003), which are processes known to be disrupted by lesions of the anterior cingulate and NAc core (Parkinson et al., 2000). Thus, in support of previous findings, the present data demonstrates that the PL is an important component of a neural circuitry that mediates the development of goal-directed responding that is distinct from an anterior cingulate- NAc core circuitry that may be involved in mediating pavlovian influences over behavior.

The acquisition of spatial context-dependent discriminative cue learning requires the successful use of spatial information within a given training session (spatial working memory), and the transfer of acquired spatial information across sessions (spatial reference memory). Our data suggest that the PL-lesioned rats showed a within-session improvement of spatial context-dependent cue learning performance that was comparable to that of the Sham and IL-lesioned groups, indicating an intact ability to retain and use spatial information to guide their discriminative approach responses within a session (not shown). However, as evidenced by their failure to enter rewarded arms over non-rewarded arms in their first three choices of arm entry at the start of each training session, PL-lesioned rats were attenuated (albeit not abolished) in the ability to recall the rewarded spatial locations *across* sessions. Thus, the present results are more in accord with the idea that the PL is important for the retention and use of incrementally acquired spatial information to guide reward-seeking, as opposed to mediating spatial working memory *per se* as has been suggested by other studies (Ragozzino et al., 1998; Kesner and Ragozzino, 2003). Rather, the present findings are somewhat consistent with the results of Seamans et al. (1995) which implicated the PL as being important in the retention and retrieval (but not acquisition) of trial-unique spatial information over a 30 min delay period. PL-inactivated rats were observed to make significant numbers of “across-phase”, as well as “within-phase” errors, indicative of their inability to use previously acquired place-reward associations in guiding and planning effective foraging strategies when the baited arms are changed.

### Opposing roles of prelimbic (PL) and infralimbic (IL) cortex in the regulation of appetitively motivated behavior

The opposite pattern of effects on spatial context-dependent discriminative cue learning generated by PL and IL lesions suggests that there may be an interaction between the PL and IL in regulating limbic control over appetitive behavior, and also that this interaction may potentially be competitive in nature. Since excitotoxic lesions of the IL led to enhanced levels of spatially discriminative cue approach behavior, it is likely that under normal circumstances, the IL exerts as an inhibitory influence upon PL function. Indeed, this notion is consistent with accumulating evidence pointing to opposing functions of the PL and IL in the control of conditioned fear and drug-seeking, with the former implicated in the initiation of conditioned fear responses and drug-seeking, and the latter in inhibitory (extinction) learning (Vidal-Gonzalez et al., 2006; Peters et al., 2008; Quirk and Mueller, 2008). Our recent work also implicates the dopaminergic innervation of the IL in opposing/dampening PL dopamine-mediated influences over appetitive spatial learning (Hayen et al., 2014). Furthermore, a recent electrophysiological finding has demonstrated highly coherent fast network oscillations in the PL and IL that disappears when the two structures are disconnected (Van Aerde et al., 2008). Intriguingly, the oscillations in the IL were found to be more powerful than that in the PL during interactions, possibly reflecting a hierarchical functional organization between the PL and IL in learning and memory processes.

In summary, the present data have revealed differential, and even opposing functional roles of the PL and IL of the mPFC in the spatial contextual control over appetitive conditioned responses. While the PL appears to be important not only in the acquisition of action-outcome associations, but also in mediating incremental learning of spatial reference memory, the IL may potentially have an important role in overriding contextual specificity, to enable context generalization under normal circumstances, in addition to its well established role in mediating stimulus-response learning. This potentially important role of IL in “downplaying” contextual influences under certain circumstances has implications for context-driven maladaptive behavior as may occur in drug-seeking and schizophrenia, and warrants further investigation.

## Author contributions

Rutsuko Ito was involved in the conception, design, data analysis and writing of this work. Rachel Ashwell was involved in the data collection, data analysis, and writing of this work. Rutsuko Ito and Rachel Ashwell agree to be accountable for all aspects of the work reported, and have approved the final version of the manuscript.

## Conflict of interest statement

The authors declare that the research was conducted in the absence of any commercial or financial relationships that could be construed as a potential conflict of interest.
